# Did the new French pay-for-performance system modify benzodiazepine prescribing practices?

**DOI:** 10.1186/1472-6963-14-301

**Published:** 2014-07-11

**Authors:** Cédric Rat, Gaëlle Penhouet, Aurélie Gaultier, Anicet Chaslerie, Jacques Pivette, Jean Michel Nguyen, Caroline Victorri-Vigneau

**Affiliations:** 1 Department of General Practice, Faculty of Medicine, 1 rue Gaston Veil, 44035 Nantes, France; 2 French National Institute of Health and Medical Research (INSERM U892)/National Center for Scientific Research (CNRS U6299), 8 quai Moncousu, 44000 Nantes, France; 3 Department of Epidemiology and Biostatistics, Nantes University Hospital, 1 place Alexis Ricordeau, 44000 Nantes, France; 4 Medical Department of the French Health Insurance System, 9 rue du Président Edouard Herriot, 44000 Nantes, France; 5 Pharmacology Department, Faculty of Medicine, 1 rue Gaston Veil, 44000 Nantes, France

**Keywords:** Benzodiazepines, Drug prescriptions, Pay-for-performance, Guideline adherence, Family practice, France

## Abstract

**Background:**

French general practitioners (GPs) were enrolled in a new payment system in January 2012. As part of a national agreement with the French National Ministry of Health, GPs were asked to decrease the proportion of patients who continued their benzodiazepine treatment 12 weeks after its initiation and to decrease the proportion of patients older than 65 who were prescribed long half-life benzodiazepines. In return, GPs could expect an extra payment of up to 490 euros per year. This study reports the evolution of the corresponding prescribing practices of French GPs during that period regarding patients who were prescribed a benzodiazepine for the first time.

**Methods:**

The national healthcare system's administrative database was used to report the longitudinal follow-up of two historical cohorts of French patients from the Pays de la Loire area.

Study patients: The “2011” and “2012” cohorts included all patients who initiated benzodiazepine regimens from April 1 to June 30 in 2011 and 2012, respectively.

The primary outcomes were the proportion of those study patients who continued benzodiazepine treatment after 12 weeks and the proportion of study patients >65 years who were prescribed long half-life benzodiazepines.

Analyses were performed using a multi-level regression.

**Results:**

In total, 41,436 and 42,042 patients initiated benzodiazepine treatment in 2011 and 2012, respectively. A total of 18.97% of patients continued treatment for more than 12 weeks in 2012, compared with 18.18% in 2011. In all, 27.43% and 28.06% of patients >65 years continued treatment beyond 12 weeks in 2011 and 2012, respectively. The *proportion* of patients >65 years who were prescribed long half-life benzodiazepines decreased from 53.5% to 48.8% (p < 0.005) due to an increase in short half-life benzodiazepine prescriptions. Patients >65 years who were prescribed *short* half-life benzodiazepines were more likely to continue treatment after 12 weeks (p < 0.005).

**Conclusions:**

Despite the pay-for-performance strategy, the number of short half-life benzodiazepine prescriptions increased between 2011 and 2012, and the number of long half-life benzodiazepine initiations remained unchanged. Reducing the proportion of long half-life benzodiazepine prescriptions might be counterproductive because prescribing short half-life benzodiazepines was associated with higher rates of continuation beyond the recommended duration.

## Background

Benzodiazepines are known to have hypnotic, anxiolytic, anticonvulsant, myorelaxant and amnesic properties. Many indications have been recognised due to benzodiazepines’ anxiolytic effects, including acute stress reactions, episodic anxiety, generalised anxiety and initial treatment for severe panic. In 2010, 15 to 20% of the French population was prescribed a benzodiazepine, which is twice as high as the percentage in other European countries [1]; thus, reducing the number of benzodiazepine prescriptions is a priority in France [2]. The extent of these prescriptions increases the number of potentially adverse effects of this drug class in the general population [3,4] and may affect mortality [5,6]. Previous research in the elderly population also demonstrated an association between benzodiazepine consumption and morbidity [3,4,6]. Moreover, Billioti de Gage recently published a cohort study demonstrating that the use of benzodiazepines in patients older than 65 was associated with an increased risk of dementia upon a 15-year follow-up [7].

Therefore, improving prescribing practices is a priority. Guidelines recommend a short-term prescription for benzodiazepines [8]; this period is limited to 2 to 4 weeks in most countries [9] and 12 weeks in France [10]. However, many publications have reported difficulties in managing benzodiazepine withdrawal in patients who became dependent because of long-term use [11-13]. Clay [1] showed that the anti-benzodiazepine campaigns initiated in most countries from 2005-2011 were unsuccessful and that the use of benzodiazepines did not decrease, despite national recommendations. Another way to limit benzodiazepine side effects might be to promote the prescription of short half-life benzodiazepines instead of long half-life benzodiazepines in patients older than 65 years [14-17]. In sum, the modification of benzodiazepine consumption in patients who have used benzodiazepines for many years remains a challenge [18].

In 2011, French policy makers speculated that a pay-for-performance intervention might motivate GPs to improve their practices. As part of a national agreement with the French National Ministry of Health and the federations of French GPs, four different priorities were defined: medical surgery organisation, quality of chronic disease management, prevention practices, and medico-economic efficiency. The overall national investment dedicated to the pay-for-performance intervention was estimated at 282 million euros [10]. Physicians were thus enrolled in this new reimbursement and payment system in January 2012 [10]. For each GP, the extra-payment package was based on a grading scale assessing 29 indicators, with a maximum of 1300 points [10]. The global extra-payment amount for each GP was estimated at 5000 euros. Benzodiazepine prescribing practices were assessed based on two indicators with a related specific extra-payment amount of 490 euros [10]. As part of the pay-for-performance intervention, GPs were asked to decrease the proportion of patients who continued their benzodiazepine treatment 12 weeks after its initiation to 12% and to decrease the proportion of patients older than 65 who were prescribed long half-life benzodiazepines to 5%.

This study reports the evolution of the prescribing practices of French GPs between 2011 and 2012 regarding patients who were prescribed a benzodiazepine for the first time. Decreases in the following indicators were expected: the proportion of patients who did not interrupt their benzodiazepine treatment 12 weeks after its initiation and the proportion of patients older than 65 years who were prescribed a long half-life benzodiazepine.

## Methods

### Design, setting, and patients

This study used the national health care system's administrative database to report the longitudinal follow-up of two historical cohorts. Access to anonymized data was provided by the National Healthcare Insurance services who participated to the study, after permission of the Healthcare Insurance authorities. All eligible patients lived on the French West Coast in the Pays de la Loire geographic area (3,571,495 inhabitants), were older than 16 years and were affiliated with one of the 1,350 GPs who practised in the geographic area at the beginning of the study (April 1, 2011).

The “2011 cohort” included all patients who had been prescribed a benzodiazepine from April 1 to June 30, 2011, and had not taken any benzodiazepines during the preceding 4 months. The “2012 cohort” included all patients who had been prescribed a benzodiazepine from April 1 to June 30, 2012, and had not taken any benzodiazepines during the preceding 4 months.

The drugs included in this study were classified as either long half-life benzodiazepines (*bromazepam, clobazam, potassium clorazepate, diazepam, ethyl loflazepate, nordazepam, prazepam, flunitrazepam* and *nitrazepam*) or short half-life benzodiazepines (*alprazolam, clotiazepam, lorazepam* and *oxazepam*) in accordance with an international classification selected by policy makers and provided to the GPs [19]. Hypnotics and “Z-drug” prescriptions (*zopiclone, zolpidem* and *zaleplon*) were not included because these drugs have a 4-week prescription limitation in France and prescribing these drugs was not a concern in the pay-for-performance experiment.

### Data collection

Benzodiazepine characteristics included the generic name, prescription dates and delivery dates. All benzodiazepine deliveries (i.e., instances of dispensing medication) recorded in the database were extracted from April 1 to June 30, 2011, and from April 1 to June 30, 2012, and the corresponding patients were identified. Other benzodiazepine deliveries were tracked for a longer period for each patient, from December 1, 2010, to August 20, 2011, and from December 1, 2011, to August 20, 2012. The proportion of patients older than 65 years who received a long half-life benzodiazepine was calculated. Only the first benzodiazepine was considered in the analysis when a patient had been successively prescribed two different benzodiazepines.

The data collected included patient characteristics, such as gender, age, socioeconomic status (characterised by specific reimbursement facilities) and two types of medical history information: diagnosis of a chronic disease (including patients benefiting from “disorder of long duration” reimbursement status) and whether a GP or a psychiatrist initiated the prescription.

### Primary outcome measures

All patients who received enough tablets to consume a benzodiazepine for a period longer than 12 weeks (based on the standard dose) were classified as “continuing patients”. The proportion of patients who did not interrupt their benzodiazepine treatment 12 weeks after its initiation (i.e., continuing patients) was calculated using the following ratio: number of continuing patients/number of patients in the cohort.

The proportion of patients who were prescribed a long half-life benzodiazepine was calculated using the following ratio: number of patients with a long half-life benzodiazepine prescription/overall number of patients with a benzodiazepine prescription.

### Statistical analysis

All analyses were performed using R 2.12.0 statistical software (R Foundation, Vienna, Austria), and Yates correction was used when required [20]. A multi-level regression analysis was used. GPs were considered as random effect, whereas patient age, sex, residency location, and socio-economic status were considered as fixed factors. An alpha level of 0.05 was chosen to assess statistical significance.

### Ethics statement

Neither ethics approval nor a specific written informed consent from participants was required in France for this retrospective database study [21].

## Results

Patient and prescriber characteristics at benzodiazepine initiation are provided in Table 1. In total, 41,436 and 42,042 patients initiated benzodiazepine treatment from April to June 2011 and April to June 2012, respectively. GPs provided more than 99% of all prescriptions in 2011 and 2012. Alprazolam was the most prescribed drug (corresponding to 41.24% of all prescribed benzodiazepine in 2011 and 43.69% in 2012), followed by bromazepam (33.31% in 2011 and 28.72% in 2012). Those patients who were prescribed two benzodiazepines during the study periods in 2011 and 2012 numbered 1,703 and 1,805, respectively.

**Table 1:** Patient and prescriber characteristics at benzodiazepine initiation (France, 2011-2012)

	**2011**		**2012**	
	**N = 41,393**		**N = 41,980**	
**Patient characteristics**	*n*	%	*n*	%
Age^a^	51.77; 51 (17.66)		52.66; 52 (17.84)	
Male	*13,696*	33.09	*14,016*	33.39
Place of residence				
Rural	*14,096*	34.05	*14,617*	34.82
Urban	*25,911*	62.60	*26,627*	63.43
Unknown	*1,386*	3.35	*736*	1.75
**Prescriber characteristics**				
Initiation by GP	*41,357*	99.91	*41,893*	99.79

^a^Mean; median (SD).

The proportion of patients who did not interrupt their benzodiazepine treatment 12 weeks after its initiation (corresponding to the first indicator) is shown in Table 2. In the overall population, 18.18% and 18.97% of patients continued the treatment for more than 12 weeks in 2011 and in 2012, respectively (p = 0.030), whereas 27.43% and 28.66% of patients older than 65 years, respectively, continued treatment beyond the 12-week period (p = 0.30).

**Table 2:** Proportion of patients who did not interrupt their benzodiazepine treatment 12 weeks after its initiation (France, 2011-2012)

	**2011**		**2012**		**p**
	**N = 41,393**		**N = 41,980**		
	**Discontinuing patients**	**Continuing patients**	**Discontinuing patients**	**Continuing patients**	
	***n*, %**	***n*, %**	***n*, %**	***n*, %**	
All patients	*33,869;* 81.82	*7,524;* 18.18	*34,018;* 81.03	*7,962;* 18.97	0.030
Patients older than 65 years	*7,180;* 72.57	*2,714;* 27.43	*7,798; 71.94*	*3,041;* 28.06	0.30

The distributions of short and long half-life benzodiazepine use in patients older than 65 years (corresponding to the second indicator) are presented in Table 3. The percentage of patients older than 65 who were prescribed a long half-life benzodiazepine decreased from 53.5% to 48.8% (p < 0.005) between 2011 and 2012.

**Table 3:** Short vs. long half-life benzodiazepines prescribed to patients older than 65 years (France, 2011-2012)

	**2011**	**2012**	
	**N = 9,894**	**N = 10,839**	**p**
	***n*; %**	***n*; %**	
Short half-life BZD^a^	4,601; 46.50	5,550; 51.20	<0.005
Clotiazepam	*118*; 1.19	*137*; 1.26	0.69
Oxazepam	*723*; 7.31	*997*; 9.20	<0.005
Lorazepam	*962*; 9.72	*1,043*; 9.62	0.83
Alprazolam	*2,798*; 28.28	*3,373*; 31.12	<0.005
Long half-life BZD^a^	5,293; 53.50	5,289; 48.80	<0.005
Bromazepam	*4,120*; 41.64	*3,907*; 36.05	<0.005
Clobazam	*115*; 1.16	*174*; 1.61	0.008
Diazepam	*64*; 0.65	189; 1.74	<0.005
Ethyl loflazepate	*112*; 1.13	*113*; 1.04	0.58
Prazepam	*624*; 6.31	*664*; 6.13	0.61
Nordazepam	*101*; 1.02	*98*; 0.90	0.43
Potassium clorazepate	*157*; 1.59	*144*; 1.33	0.13

^a^Benzodiazepine.

Table 4 synthesises the results of the two previous indicators. This table shows that patients older than 65 years who were prescribed a short half-life benzodiazepine were more likely to continue the treatment beyond the 12-week limit compared with those prescribed a long half-life benzodiazepine (p < 0.005).

**Table 4:** Association between benzodiazepine discontinuation and drug half-life in patients older than 65 years

	**Short half-life benzodiazepine**	**Long half-life benzodiazepine**	**p**
**2011**	N = 4,601	N = 5,293	
Continuing patients (n; %)	*1,425*; 30.97	*1,289*; 24.35	<0.005
**2012**	N = 5,550	N = 5,289	
Continuing patients (n; %)	*1,755*; 31.62	*1,286*; 24.31	<0.005

## Discussion

### Main findings

The proportion of patients who continued their benzodiazepine prescriptions beyond the recommended duration did not decrease between 2011 and 2012, despite recommendations and financial incentives. On the contrary, this database study shows a slight but significant increase in the number of patients who did not interrupt benzodiazepine consumption. One in five patients who initiated a benzodiazepine regimen continued drug intake beyond the recommended 12-week duration, which increased to more than one in four patients over the age of 65. The *proportion* of long half-life benzodiazepine prescriptions decreased in the latter population, which could be attributed to a 20% increase from 2011 to 2012 in the overall prescription of short half-life benzodiazepines, compared with no change in long half-life benzodiazepine prescription. This study shows that substituting long half-life benzodiazepines with short half-life benzodiazepines might be counterproductive because the prescription of short half-life benzodiazepines was significantly associated with treatment continuation beyond the recommended duration.

### Strengths and weaknesses

The pay-for-performance intervention that was evaluated in this study was implemented as a nationwide strategy in a country in which these drugs are extensively prescribed. Policy makers and GPs organisations selected the objectives and the related outcomes of their own initiatives. Our study was designed to be consistent with the objectives and evaluations put forth by policy makers, and the research findings should be relevant to GPs in clinical practice.

The study has several limitations because policy makers primarily designed the implemented intervention without researchers’ opinions. This pay-for-performance study was an uncontrolled before-and-after study, which did not allow the assertion of a causal link between the intervention and the observed changes [22,23]. An optional pay-for-performance system had been piloted in France previously; consequently, the effective novelty of the pay-for-performance scheme probably concerned only two-thirds of the GPs who participated to the study. Information in this study was extracted from large databases derived from healthcare insurance systems, which is similar to previous studies [24-26]. A limitation reported in other studies of inappropriate prescribing was that information about disease and indications could not be considered. Drug intake could be assessed using only proxy measures because data collection was based on reimbursement.

### Findings relative to other studies

The positive impact of financial incentives on benzodiazepine prescribing practices is difficult to assess. Our results are consistent with previous evaluations of the effectiveness of pay-for-performance strategies. Evidence of improvement in patient health is lacking [22,23]. Flodgren et al. reported that financial incentives for physicians were generally ineffective for improving compliance with guideline outcomes [23]. In contrast, two recent Dutch studies demonstrated a link between payment facilities and benzodiazepine use [27,28], although the interventions in these studies most likely had a greater impact on patient behaviour than on GPs’ prescribing practices. In particular, these studies evaluated the impact of benzodiazepine delisting by health insurance. The first study focused on indications for “anxiety” and “sleep disorders” and demonstrated a moderate impact of delisting on the number of benzodiazepine treatment initiations [27]. The second study compared the number of days that each patient underwent benzodiazepine treatment during the 2 years before and 2 years after delisting. The number of days of treatment decreased, especially in patients with initial low intake [28].

Financial incentives for GPs did not favour the discontinuation of benzodiazepine prescribing. Two interpretations of this result must be considered. First, the inappropriate practices of GPs are likely not due to a lack of motivation. Previous studies have also shown that GPs are aware of their actions [29]. Thus, further interventions should focus on other solutions. For instance, cognitive behavioural therapies are recommended [30], but no reimbursement is provided to the patient for such therapies, even if he or she consults a psychologist [31]. Second, patients with psychological disorders are likely to face difficulties that require sustained long-term care. Karanikolos reported that the prevalence of mental health disorders in people undergoing primary care increased significantly in European countries in association with the current economic crisis and austerity policies [32]. Many anxiety and depressive symptoms can be attributed to either individual or family unemployment or difficulties with payments [33]. Many recent publications have also reported increasing suicide rates in European countries [34-36], so the study periods were unfavourable for expectations of a decrease in benzodiazepine consumption. In further studies, clinical assessment of indications and distinctions among anxiety, sleep disorders and other indications would facilitate an improved focus on inappropriate long-term use of benzodiazepines. GPs should reconsider treatment indications to shift towards non-pharmacological treatments or other drugs, such as serotonin reuptake inhibitors, to resolve this issue regarding benzodiazepine prescriptions. These alternative treatments could help to avoid the side effects of benzodiazepines.

The reduced proportion of long half-life benzodiazepine prescriptions was consistent with the key message of policy makers to GPs. Previous authors suggested that reducing the use of long half-life benzodiazepines in individuals older than 65 years could reduce the risk of sedation, falls, hip fractures, memory disorders and accidents [37,38]. However, other publications did not find the same associations [39,40]. The use of short-acting benzodiazepines has also been associated with fall-related injuries [41]. Therefore, the changes in physician practice that were observed in this study might not be relevant. Prescribers should evaluate the indication, dose and duration of benzodiazepine treatment according to the clinical characteristics of patients. Half-life duration is an important consideration but should not be the main reason for choosing a benzodiazepine. Indeed, half-life benzodiazepine classifications differ between different authors. The French pay-for-performance intervention refers to an international classification published by Laroche in 2007 [42], but other studies distinguish three types of benzodiazepines: short, intermediate and long half-life benzodiazepines [43]. Last but not least, this study suggests that the use of short half-life drugs might increase the risk of addiction, which is consistent with their pharmacology [44].

### Implications for clinicians and policy makers

This study emphasises the difficulties that clinicians face in anxiety management. A key message is that substitution of a long half-life benzodiazepine with a short half-life one is likely suboptimal. A better substitution might be the use of antidepressants rather than benzodiazepines for long-term treatment [45]. Our study also demonstrated the limited impact of the pay-for-performance system on anxiety management practices in primary care. A new approach might be the transfer of part of the amount reserved for the pay-for-performance system to reimbursement for psychologist consultations in France [45].

## Conclusions

The implementation of the pay-for-performance strategy did not affect the prescription of long half-life benzodiazepines, while the number of prescriptions of short half-life drugs increased between 2011 and 2012. An adverse effect of this evolution was the continuation of benzodiazepine treatments for more than 12 weeks, insofar as short half-life drugs have been associated with a higher rate of withdrawal than long half-life drugs.

## Competing interests

The authors declare that they have no competing interests.

## Authors’ contributions

CR conceived of the study, participated in its design and supervision and helped to draft the manuscript. GP participated in the design of the study, participated in data extraction, and helped to draft the manuscript. AG performed the statistical analysis and helped to draft the manuscript. AC and JP participated in the design, were responsible for data extraction, and provided administrative or technical support. JMN participated in the design of the study, and was responsible for the statistical analysis. CVV participated to study supervision and helped to draft the manuscript. All authors read and approved the final manuscript.

## Pre-publication history

The pre-publication history for this paper can be accessed here:

http://www.biomedcentral.com/1472-6963/14/301/prepub
